# Matrix Metalloproteinase-7 Promoter Site Single Nucleotide Polymorphism (-181A>G) in Epithelial Ovarian Cancer in the Eastern Indian Population

**DOI:** 10.7759/cureus.56417

**Published:** 2024-03-18

**Authors:** Anudeep P.P., Suchitra Kumari, Saroj Dasmajumdar, Manaswini Mangaraj

**Affiliations:** 1 Biochemistry, All India Institute of Medical Sciences, Bhubaneswar, Bhubaneswar, IND; 2 Radiation Oncology, All India Institute of Medical Sciences, Bhubaneswar, Bhubaneswar, IND

**Keywords:** extracellular matrix, single nucleotide polymorphism, ovarian cancer, matrix metalloproteinase 7, genotype

## Abstract

Background: Matrix metalloproteinase-7 (MMP7) plays multiple roles in different stages of tumor development. Elevated MMP7 activity has been reported in ovarian cancer. Single nucleotide polymorphism (SNP) of promoter sites of the MMP7 gene has been shown to cause alteration in gene expression, hence resulting in changes in susceptibility to various diseases and tumor development.

Methods: The current study evaluated the association of epithelial ovarian cancer risk with MMP7 promoter site -181A>G polymorphism in the population of eastern India. The present case-control study included 64 histopathologically confirmed cases of epithelial ovarian cancer and 100 control subjects. The MMP7 -181A/G polymorphism was identified using polymerase chain reaction-restriction fragment length polymorphism. The association between genotypes and epithelial ovarian cancer risk was analyzed by odds ratio (OR) with a 95% confidence interval.

Results: The frequencies of AA, AG, and GG genotypes in ovarian cancer cases were 37.5%, 46.9%, and 15.6%, respectively, while that of control subjects were 56%, 36%, and 8%, respectively, in the study population. By taking the wild-type AA genotype as a reference, it was found that genotype GG was associated with a significant risk for epithelial ovarian cancer (OR: 2.92). Frequency distribution of genotypes did not show any significant association with tumor characteristics like the International Federation of Gynecology and Obstetrics (FIGO) stage, histology, lymph node status, and distant metastasis.

Conclusion: The present study demonstrated the association of MMP7 promoter site -181 GG genotype and the G allele with increased risk for epithelial ovarian cancer in the eastern Indian population.

## Introduction

Ovarian cancer is the eighth most common cancer among women in the world [1]. In 2018, the incidence of ovarian cancer in India was 36,170, and death due to ovarian cancer was 24,015 [2]. Even with vast advancements in the treatment of ovarian carcinoma, it still remains the second most common cause of death among gynecological malignancies in the world. Due to anatomical position, complicated histopathology, nonspecific symptoms, and late presentations, ovarian neoplasms are diagnosed usually in late stages [3]. Despite intensive research in this field, effective screening tools are still not available to identify the disease at early stages.

The ability of tumor cells to invade the extracellular matrix (ECM) and access the circulation plays a key role in seeding the metastasis. ECM is a highly organized dynamic non-cellular structure that undergoes constant remodeling in the body. The balance between synthesis and degradation of ECM should be maintained for normal integrity of tissue. There is a constant interaction taking place between the tumor and its microenvironment. Dysregulation of ECM remodeling has been reported during tumor development [4]. Matrix metalloproteinases (MMPs) are a family of zinc-dependent endopeptidases. The main function of MMPs is to degrade the ECM. Finely orchestrated activity of MMPs is essential for various physiological processes like wound repair, bone growth, tissue remodeling, embryonic development, and inflammatory responses, while dysregulation of matrix metalloproteinase-7 (MMP7) activity is indicated in various pathological processes [5].

In the MMP family, MMP7 (matrilysin-1) is the smallest member and can degrade various ECM substrates, including type IV collagen, elastin, and fibronectin. They can also cleave various non-matrix substrates of cell surface like Fas ligand, pro-tumor necrosis factor, and E-cadherin [6]. MMP7 can play diverse roles during the development of tumors. Previous studies have demonstrated the action of MMP7 on the development of apoptosis resistance, tumor growth, angiogenesis, and cancer cell invasion [7-9]. Elevated MMP7 is described in various cancers, including ovarian cancers [10,11]. Gene encoding MMP7 is located on chromosome 11q21-q22. Single nucleotide polymorphism (SNP) of promoter sites of the MMP gene has been shown to cause alteration in gene expression, hence resulting in changes in susceptibility to various diseases and tumor development [6]. Two widely studied SNPs in the promoter sites of MMP7 genes are -181A>G (rs11568818) and -153 C>T (rs2285053) [12]. G allele at MMP7 promoter site -181A>G has been found to increase promoter activity two to three times, resulting in increased MMP7 expression [13]. While some studies have shown a positive association between the MMP7 -181A>G polymorphism and tumor development, others did not find any significant association [14-16]. A risk allele frequency may differ between populations, and an allele may interact with other genetic or environmental factors that differ as per the demographic location. Thus, the association of polymorphism with cancer risk can vary in different populations [17].

Understanding the genetic determinants of ovarian cancer may prove helpful in predicting the aggressiveness and prognosis of the disease. There is only a limited number of studies regarding the role of MMP7 -181A>G (rs11568818) polymorphism in the development of ovarian cancer [18]. The published data based on an Indian study to show the association of the MMP7 promoter site SNP (181A>G) with the development of ovarian cancer is still lacking. A thorough understanding of the association between MMP7 SNP and ovarian cancer may help to unveil the role of MMP7 in the etiology of ovarian cancer. This may help to develop a genetic marker for the identification of ovarian cancer risk as well as to facilitate the treatment in personalized medicine. The potential of MMP7 as a genetic marker was presented as a thesis article, and the present study was conducted to evaluate the association of SNP (-181A>G) of the promoter site of MMP7 in epithelial ovarian cancer patients of the population of eastern India.

## Materials and methods

Study population

The present case-control study was conducted in the Molecular Biology Laboratory, Department of Biochemistry, All India Institute of Medical Sciences (AIIMS), Bhubaneswar, Odisha, India. Sixty-four subjects attending the outpatient clinic of the Obstetrics and Gynecology Department and Radiation Oncology Department, clinically diagnosed, histopathologically confirmed for epithelial ovarian cancer, and within the age group of 45-65 years were enrolled as cases in the study. Age-matched, 100 females, resident location matched (urban/rural), and apparently healthy individuals were enrolled as controls. All benign ovarian tumors and secondary ovarian cancers were excluded from the study. This study was approved by the institute ethics review board, i.e., Institute Ethics Committee, AIIMS Bhubaneswar (Reference Number: IEC/AIIMS BBSR/PG Thesis/2019-20/18), and samples were collected after informed written consent was taken. Clinicodemographic details were obtained as per the questionnaire prepared and self-reported responses of the participants were recorded. Staging of ovarian carcinoma was done by using the International Federation of Gynecology and Obstetrics (FIGO) guidelines [18].

Methods

**Figure 1:**
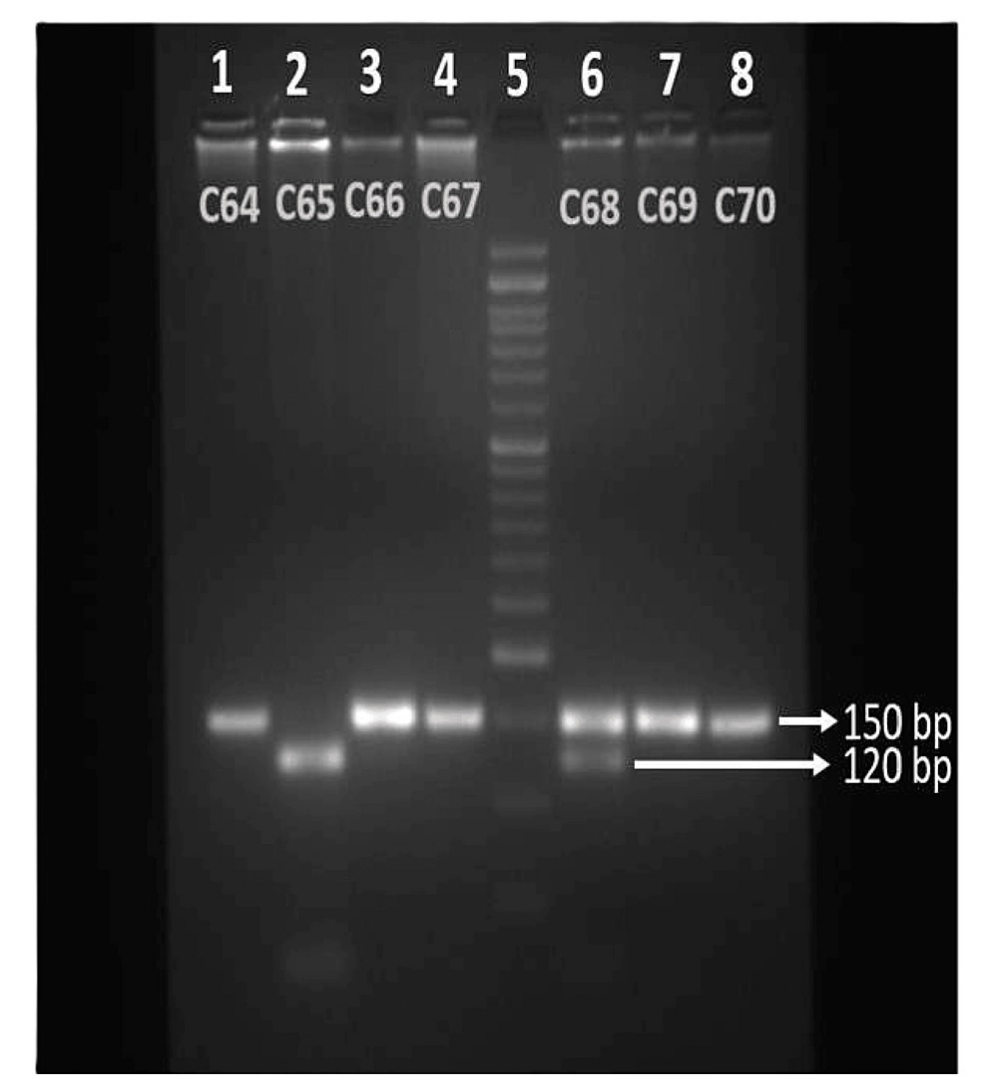
Agarose gel picture of RFLP products of MMP7 (181A>G) genotypes. Lanes 1, 3, 4, 7, 9 - homozygous AA (150 bp). Lane 2 - homozygous GG (120 bp). Lane 6 - heterozygous AG (150 bp/120 bp). Lane 5 - 50 bp DNA ladder. RFLP: restriction fragment length polymorphism; MMP7: matrix metalloproteinase-7.

Venous blood samples (3 ml) were collected and kept in an ethylenediaminetetraacetic acid (EDTA) vacutainer for MMP7 gene polymorphism studies. All experiments were performed in accordance with relevant guidelines and regulations. For detection of MMP7 -181A>G (rs11568818) SNP, DNA was extracted from blood samples using a commercially available spin column method (QIAamp DNA Blood Kit, cat no. 51106, Qiagen, Hilden, Germany). The quality of the DNA was evaluated by Nano Bio spectrophotometer (Analytical Technologies Limited, Vadodara, India) and polymerase chain reaction (PCR) amplification was done using primers spanning the 181A>G (rs11568818) region of the MMP7 gene using forward primer sequence 5’-TGG TAC CAT AAT GTC CTG AAT G-3’ and the reverse primer sequence 5’-TCG TTA TTG GCA GGA AGC ACA CAA TGA ATT-3’15. PCR was performed in a total volume of 25 μl using 10 p mol of forward primer, 10 p mol of reverse primer, 100 ng extracted DNA, and PCR master mix (DreamTaq^TM^ Hot Start PCR Master Mix, Thermo Fisher Scientific, Waltham, CA). The PCR conditions include initial denaturation at 94°C for three minutes, followed by denaturation at 94°C for 30 seconds, annealing at 56°C for 30 seconds, and extension at 72°C for 30 seconds over a period of 40 cycles with final extension at 72°C for 10 minutes. The PCR product was visualized by running in a 2% agarose gel stained in ethidium bromide and the image was documented in a gel documentation system. The restriction fragment length polymorphism (RFLP) method was used to analyze rs11568818 polymorphism. As per kit specifications, the amplified PCR product was digested overnight at 37°C with 10 units of restriction endonuclease enzyme, i.e., EcoR1 10 U/µL (Thermo Fisher Scientific, Waltham, CA) in a 30 μL reaction volume along with enzyme buffer. The digested PCR product was separated on 3% agarose gel stained with ethidium bromide and visualized under a gel documentation system (Syngene Chemiluminescence, Cambridge, UK). An uncut fragment at 150 bp was interpreted as homozygous A alleles, a combination of 150, 120, and 30 bp for heterozygous genotypes and DNA bands with sizes at 120 and 30 bp for the homozygous G allele. Gel pictures are interpreted and documented as an allelic profile of each subject (Figure 1). To confirm MMP7 genotype results, 20% of the PCR amplified sample was selected randomly and repeated for restriction digestion and analyzed using agarose gel electrophoresis.

Statistical analysis

Statistical analysis was done using IBM SPSS version 20 (IBM Corp., Armonk, NY). Comparisons of the demographic data between the case and control groups were done using the Student’s t-test and χ2 test. The Hardy-Weinberg equilibrium was tested in cases and control groups. Frequencies of the genotypes and alleles of the MMP7 -181A>G (rs11568818) polymorphism were compared between the case and controls using the chi-square test. The associations between MMP-7 genotypes and ovarian cancer risk were assessed using the odds ratio (OR) with a 95% confidence interval. Cases were stratified into different groups based on disease variables and the distribution of frequency between the groups was compared using the chi-square test or Fisher’s exact test. A p-value of less than 0.05 was considered statistically significant.

## Results

Demographic and clinical characteristics of epithelial ovarian cancer patients and controls

The current study included 64 epithelial ovarian cancer patients and 100 control subjects. Selected demographic and clinical characteristics of cases and control subjects are listed in Table 1. There was no statistically significant difference between the case and control groups in age. Most of the ovarian cancer cases were postmenopausal women. There was no statistically significant difference between the groups in menopausal status. The body mass index (BMI) of controls was significantly higher compared to cases. Neither age of menarche nor parity status showed any significant difference between the groups. There was no statistically significant difference between the case and control groups regarding the history of oral contraceptive pills (OCP) consumption, history of polycystic ovary syndrome (PCOS), and family history of cancer.

**Table 1:** Demographic and clinical characteristics of epithelial ovarian cancer patients and controls. Data are presented as mean ± SD or N (%). * Statistically significant p < 0.05 vs. controls. PCOS: polycystic ovary syndrome; OCP: oral contraceptive pills.

Characteristics	Case (64)	Control (100)	P-value
Age (years)	51.98 ± 8.25	50.03 ± 10.52	0.187
Height (cm)	156.05 ± 5.08	161.42 ± 6.8	<0.001*
Weight (kg)	51.06 ± 10.41	64.51 ± 15.37	<0.001*
BMI (kg/m2)	20.95 ± 4.04	24.77 ± 5.80	<0.001*
Age at menarche (years)	14.3 ± 0.90	14.7 ± 1.10	0.425
Menopausal history			
Premenopausal	12 (18.8%)	22 (22%)	0.616
Postmenopausal	52 (81.3%)	78 (78%)	
Parity			
Nulliparous	6 (9.3%)	8 (8%)	0.952
Uniparous	3 (4.7%)	5 (5%)	
Multiparous	55 (85.94%)	87 (87%)	
History of PCOS			
No	62 (96.9%)	98 (98%)	0.644
Yes	2 (3.1%)	2 (2%)	
History of OCP consumption			
No	58 (90.6%)	85 (85%)	0.293
Yes	6 (9.4%)	15 (15%)	
Family history of cancer			
No	57 (89.1%)	86 (86%)	0.567
Yes	7 (10.9%)	14 (14%)	

Clinical characters of epithelial ovarian cancer cases are represented in Table 2. Most of the tumors were serous carcinoma type, i.e., 42 (65.6%). Most of the cases of ovarian cancer were of the FIGO stage III group, i.e., 33 (51.56%). Since most of the cases were of advanced FIGO stages, most of them had nodal metastasis by the time of diagnosis, i.e., 41 (64.1%).

**Table 2:** Clinical characteristics of the cases of epithelial ovarian cancer. Data are presented as N (%). FIGO: International Federation of Gynecology and Obstetrics.

Characteristics	Cases (64), N (%)
Histology	
Serous	42 (65.6)
Endometrioid	7 (11)
Mucinous	7 (11)
Clear cell	6 (9.3)
Others	2 (3.1)
FIGO stage	
I	8 (12.5)
II	8 (12.5)
III	33 (51.56)
IV	15 (23.4)
Lymph node metastasis	
No	21 (32.8)
Yes	41 (64.1)
Unknown	2 (3.12)
Distant metastasis	
No	35 (54.7)
Yes	16 (25)
Unknown	13 (20.3)

Association of MMP7 (-181A>G) polymorphism with susceptibility to epithelial ovarian cancer

The frequencies of AA, AG, and GG genotypes in ovarian cancer cases and control subjects are represented in Table 3. In the study population, the MMP7 (-181A/G) SNP was found at a minor allele frequency (MAF) of 0.30 (MAF of case = 0.39 and control = 0.27). The genotypic frequencies in cases and controls were in Hardy-Weinberg equilibrium (cases: χ2, p = 0.94; control: χ2, p = 0.44).

**Table 3:** Association of MMP7 (-181A>G) polymorphism with susceptibility to epithelial ovarian cancer. MMP7: matrix metalloproteinase-7; OR: odds ratio; CI: confidence interval, N (%). * Statistically significant p < 0.05 vs. reference.

Genotype	Case, N (%) (N = 64)	Control, N (%) (N = 100)	OR	95% CI	P-value
AA	24 (37.5)	56 (56)	1 (Ref)	NA	NA
AG	30 (46.9)	36 (36)	1.94	0.98-3.84	0.054
GG	10 (15.6)	8 (8)	2.92	1.03-8.30	0.04*
Dominant model					
AA	24 (37.5)	56 (56)	1 (Ref)	NA	NA
AG+GG	40 (62.5)	44 (44)	2.12	1.17-4.03	0.021*
Recessive model					
AA+AG	54 (84)	92 (92)	1 (Ref)	NA	NA
GG	10 (16)	8 (8)	2.13	0.79-5.72	0.128
Allele					
A allele	78 (60.9)	148 (73.5)	1 (Ref)	NA	NA
G allele	50 (39.1)	52 (27.5)	1.82	1.13-2.93	0.013*

When compared to the control group, the GG genotype frequency was greater in the cases. By taking the wild-type AA genotype as a reference, it was found that genotype GG was associated with a significant risk for epithelial ovarian cancer (OR: 2.92, p = 0.04). Though the AG genotype was also associated with an increased risk for ovarian cancer in the study population, it was not found to be statistically significant (OR: 1.94, p = 0.054). The link between the MMP7 -181A/G polymorphism and the risk of ovarian cancer was also statistically significant in the dominant model (AG+GG vs. AA) (OR: 2.12, p = 0.021). When comparing individuals with the G allele at the MMP7 promoter site -181A/G to those with the wild-type A allele, the OR was 1.82 (p = 0.013).

Distribution of MMP7 (-181A/G) polymorphism in epithelial ovarian cancer, stratified according to different disease variables

The association of the frequency distribution of genotypes and tumor characteristics like FIGO stage, histology, lymph node status, and distant metastasis was also evaluated (Table 4). Most of the patients in early and late FIGO stages were of AG genotype. There was no significant difference in the frequency of the -181A/G polymorphism in patients with different FIGO stages. When compared between early (I+II) and late (III+IV) FIGO stages, the AG genotype was associated with an OR of 1.09, but it was statistically insignificant. GG genotype also did not show any increased risk for the late FIGO stage. AG genotype did not show any significant risk for lymph node or distant metastasis compared to the wild-type AA genotype. However, the GG genotype had an odds ratio of 1.88 for distant metastasis when compared to the wild-type AA genotype, which was not statistically significant. Compared to serous type carcinoma, other epithelial ovarian type carcinoma did not show any significant risk with respect to genotype distribution.

**Table 4:** Frequency distribution of MMP7 (-181A/G) polymorphism in epithelial ovarian cancer, stratified according to different disease variables. Data are represented as N (%) or odds ratio (95% confidence interval). MMP7: matrix metalloproteinase-7; FIGO: International Federation of Gynecology and Obstetrics.

Variable	Frequency	Frequency	OR (95% CI)	P-value
FIGO stage	FIGO stage I+II (16), N (%)	FIGO stage III+IV (48), N (%)		
AA	6 (38)	18 (38)	1 (Ref)	NA
AG	7 (44)	23 (48)	1.09 (0.31-3.83)	0.887
GG	3 (19)	7 (15)	0.78 (0.15-4.00)	1.000
Histology	Serous (42)	Others (22)		
AA	17 (40)	7 (32)	1 (Ref)	NA
AG	19 (45)	11 (50)	1.41 (0.44-4.45)	0.772
GG	6 (15)	4 (18)	1.62 (0.35-7.56)	0.692
Lymph node status	Involved (21)	Not involved (41)		
AA	7 (33)	16 (39)	1 (Ref)	NA
AG	11 (52)	18 (44)	0.72 (0.22-2.29)	0.573
GG	3 (15)	7 (17)	1.02 (0.20-5.14)	1.000
Distant metastasis	Involved (35)	Not involved (16)		
AA	14 (40)	5 (31)	1 (Ref)	NA
AG	15 (43)	7 (44)	1.31 (0.34-5.09)	0.699
GG	6 (17)	4 (25)	1.88 (0.37-9.48)	0.675

## Discussion

The present study has been conducted to evaluate the association of SNP (181A>G) of the promoter site of the MMP7 (rs1168818) gene with the risk of epithelial ovarian cancer.

Demographic and clinical characteristics of epithelial ovarian cancer patients and control subjects

Ovarian cancer is primarily considered a disease of postmenopausal women. The mean age of ovarian cancer cases in the present study was 51.98 years. The mean age of diagnosis, according to prior studies, is around 50-79 years old [19]. Most of the ovarian cancer patients in the current study were post-menopausal women. In the present study, compared to cases, BMI was higher in the control group. The association between ovarian cancer risk and being overweight is controversial. A meta-analysis reported a higher risk of ovarian cancer with increased body weight. Extreme obesity in particular had a higher risk. This effect was found to be significant in premenopausal women only [20]. As per the incessant ovulation hypothesis, the early age of menarche and the late age of menopause via increasing the number of ovulatory cycles enhance the risk of ovarian cancer [21]. However, the current study did not find any significant difference in the age of menarche between the case and control groups. Most women in both study groups were multiparous, hence we did not find any significant difference in parity status between cases and controls. Studies have shown the protective effect of pregnancy against ovarian cancer risk. A retrospective cohort study in the Asian population demonstrated lower ovarian cancer risk with increasing parity [22]. Most of the women did not have a history of OCP consumption in both groups. The current study did not find any significant difference in OCP consumption between cases and controls. Previous research studies have reported a reduction in ovarian cancer risk with the consumption of OCP [23]. Among epithelial ovarian cancer included in the current study, the majority belongs to the serous type, i.e., 42 (65%), as expected. The late FIGO stage group, which includes FIGO stage III & IV, had more patients, i.e., 48 (75% of cases), compared to the early FIGO stage group (FIGO stage I & II). Because ovarian cancer is typically asymptomatic in its early stages, the majority of cases were detected at advanced stages, resulting in a disparity in FIGO stage distribution of ovarian cancer cases. Most of the recruited ovarian cancer cases had nodal metastasis at the time of diagnosis. Of the cases, 25% had distant metastasis also. This points out the delay in the diagnosis of ovarian cancer, where most cases end up with metastasis by the time of diagnosis.

Association of MMP7 (-181A>G) polymorphism with susceptibility to epithelial ovarian cancer

The current study used the PCR-RFLP method to find out the SNP at the -181 position of MMP7 in the population of Eastern India. The present study reported a minor (G) allelic frequency (MAF) of the case (0.39) and control (0.27) groups. Kesh et al. evaluated the association of gastric carcinoma risk with MMP7 -181A/G SNP in the eastern Indian population and reported an MAF of 0.35 for this particular SNP [24].

In the present study, the GG genotype was found to be associated with a 2.92-fold higher risk for epithelial ovarian cancer compared to the wild-type AA genotype. Considering the allele-specific risk, the G allele at the -181 position of the MMP7 promoter region conferred a 1.82 times higher risk for epithelial ovarian cancer. To the best of our knowledge, there is no published data on the association between MMP7 -181A/G (rs11568818) and ovarian cancer risk in an Indian population. Susceptibility to ovarian cancer and MMP7 SNP has been investigated previously in the North China population. Li et al. studied MMP7 -181A/G and documented significantly increased susceptibility to ovarian cancer (OR: 3.53) with -181G allele (A/G + G/G) compared to the A/A genotype. Our study also documented a similar increased risk for ovarian cancer, though OR was slightly less (1.82) in comparison to the study by Li et al. [18].

In the Indian population, MMP7 (-181A>G) polymorphism was previously evaluated to probe its association with various other cancers. Singh et al. reported an OR of 1.99 associated with the GG genotype in comparison to the AA genotype for cervical cancer risk in the Indian population [25]. A study that evaluated gastric cancer risk with MMP7 SNP in the Eastern Indian population also reported a higher risk associated with the GG genotype [24]. Similarly, in the Kashmiri population, Malik et al. also found a higher risk for the development of esophageal cancer in people with the GG genotype at the -181 position of the MMP7 promoter site [26]. The north Indian population was evaluated for bladder cancer by Srivastava et al. in the year 2010, and it was observed that the MMP7 -181 GG genotype conferred a 2.38-fold risk in comparison to wild-type AA [27]. All these observations suggest that the MMP7 (-181A>G) genetic polymorphism may contribute to the susceptibility of cancer. This SNP in the promoter site of MMP7 may act by generating or obliterating binding sites for transcriptional factors resulting in the altered transcriptional activity of MMP7 [28]. Further studies are necessary to evaluate the mechanism behind the allele-specific effect of this polymorphism on ovarian cancer risk.

Frequency distribution of MMP7 (-181A/G) polymorphism in epithelial ovarian cancer, stratified according to different disease variables

The present study did not find any correlation between the FIGO stages and genotype distribution. AG or GG genotype was not found to exert any significant additional risk for late-stage ovarian cancer. Nodal or distal metastasis also did not show any significant association with genotype distribution. A previous study by Banday et al. showed that colorectal cancer patients with the GG genotype of the MMP7 -181A/G SNP had a higher risk of lymph node invasion in the Kashmiri population [29].

Though our study showed increased ovarian cancer risk with AG and GG genotypes, we did not find any significant association between AG or GG genotypes with advanced ovarian cancer stages. The present study also did not find any evidence of a link between genotype distribution and tumor histology. Limited sample size and disparity in the distribution of cases in FIGO stages in the present study might have impacted the results. Besides these limitations, the serum estimation of MMP7 could give us an insight into the expression of this gene and further substantiate the role of MMP7 in ovarian cancer. Functional assays revealing tissue expression will relate the genotypes with phenotype heterogeneity.

## Conclusions

The present study demonstrated the association of MMP7 promoter site (-181A>G) polymorphism in epithelial ovarian cancer patients in the eastern Indian population. MMP7 promoter site -181 GG genotype and the G allele were found to have an increased risk for epithelial ovarian cancer. However, further population-based research with a larger sample size needs to be conducted to ascertain its clinical utility.
